# Combining single-cell sequencing data to construct a prognostic signature to predict survival, immune microenvironment, and immunotherapy response in gastric cancer patients

**DOI:** 10.3389/fimmu.2022.1018413

**Published:** 2022-10-10

**Authors:** Bo Hu, Yan Meng, Chao Qu, Bing-Yan Wang, Dian-Rong Xiu

**Affiliations:** Department of General Surgery, Peking University Third Hospital, Beijing, China

**Keywords:** gastric cancer, single-cell sequencing, immune microenvironment, immunotherapy, prognosis

## Abstract

**Background and objective:**

Gastric cancer (GC) represents a major factor inducing global cancer-associated deaths, but specific biomarkers and therapeutic targets for GC are lacking at present. Therefore, the present work focused on developing an immune-related genetic signature at the single-cell level for categorizing GC cases and predicting patient prognostic outcome, immune status as well as treatment response.

**Methods:**

Single-cell RNA-sequencing (scRNA-seq) data were combined with bulk RNA-seq data in GC patients for subsequent analyses. Differences in overall survival (OS), genomic alterations, immune status, together with estimated immunotherapeutic outcomes were measured between different groups.

**Results:**

Nine cell types were identified by analyzing scRNA-seq data from GC patients, and marker genes of immune cells were also selected for subsequent analysis. In addition, an immune-related signature was established to predict OS while validating the prediction power for GC patients. Afterwards, a nomogram with high accuracy was constructed for improving our constructed signature’s clinical utility. The low-risk group was featured by high tumor mutation burden (TMB), increased immune activation, and microsatellite instability-high (MSI-H), which were related to the prolonged OS and used in immunotherapy. By contrast, high-risk group was associated with microsatellite stability (MSS), low TMB and immunosuppression, which might be more suitable for targeted therapy. Meanwhile, the risk score generated by our signature was markedly related to the cancer stem cell (CSC) index. In addition, the immunotherapeutic response prediction accuracy of our signature was validated in an external dataset IMvigor210 cohort.

**Conclusion:**

A signature was constructed according to scRNA-seq data analysis. The signature-screened low- and high-risk patients had different prognoses, immune statuses and enriched functions and pathways. Such results shed more lights on immune status of GC, prognosis assessment, and development of efficient immunotherapeutic treatments.

## Introduction

Gastric cancer (GC) is a major factor inducing cancer-associated mortality globally, which causes over 700,000 death cases annually (1). Currently, early-stage GC is mainly treated with endoscopic resection (2), while surgery is mainly adopted for resectable GC at the intermediate or late stage, including D2 lymphadenectomy. Patients with advanced cancer can gain benefits from adjuvant or perioperative radiotherapy and chemotherapy (3, 4). In most cases, however, GC is advanced at the time of diagnosis, resulting in the dismal patient survival although surgical and medical treatments have greatly improved. For advanced GC, its median survival time remains as low as 12-15 months (5), and novel therapies need to be introduced.

Nowadays, cancer immunotherapy is becoming the robust and candidate clinical option to treat cancer, and major achievements have been made in breast cancer (BC) (6), prostate cancer (PC) (7) and melanoma (8). Immune checkpoint inhibitors (ICIs), the novel treatment standards of different cancers like GC, have demonstrated promising clinical benefits in several populations (9–11). Nevertheless, the response of immunotherapy in GC has been frustrating overall to date, as current methods are often ineffective on stimulating immunity and tumors continue to grow even though a measurable immune response is measured (12). Despite the histopathology or molecular subtype, GC is not the separate cancerous epithelial cell block. In contrast, GC tumor has complicated morphology, and tumor cells are surrounded *via* the cellular environment referred to as the tumor microenvironment (TME), which contains multiple types of cells like immune cells, endothelial cells, and fibroblasts. Therefore, exploring GC immune landscape from different aspects, investigating its immune characteristics, and developing approaches for the accurate prediction of immune status as well as immunotherapeutic response in GC are of great importance.

The emergence of single-cell RNA-sequencing (scRNA-seq) has offered a great chance to explore single-cell gene profiling data (13). ScRNA-seq becomes the promising alternative to investigate critical biological issues such as cellular heterogeneity. In terms of cancer research, intra-tumor heterogeneity is a critical challenge encountered by precision cancer treatment. scRNA-seq evolution can offer statistical significance for characterizing different cell subsets in cancers. This work analyzed the tumor immune microenvironment (TIME) of GC from the novel perspective, starting from the single-cell level and combining bulk transcriptomic data. Furthermore, an immune-related gene (IRG)-based riskscore model was also constructed for evaluating different immune statuses and therapeutic responses among high- and low-risk cases. Our study can shed more lights on exploring the mechanisms related to diverse immunotherapeutic responses among GC cases and offer new insights into immunotherapeutic strategies for GC.

## Materials and methods

### Data extraction and mRNA profile mining

Expression pattern of class 3 messenger RNA (mRNA) of GC (fragments per kilobase million, FPKM), along with relevant clinical information, was obtained in Gene Expression Omnibus (GEO) (https://www.ncbi.nlm.nih.gov/geo/) and The Cancer Genome Atlas (TCGA) (https://cancergenome.nih.gov) databases. Besides, this work also acquired TCGA-stomach adenocarcinoma (STAD), GSE84437, GSE62254 and GSE15459 datasets in later analyses, where the latter two were employed for external validation. Immune subtypes were acquired based on TCGA-derived pan-cancer information, which were later adopted for examining relationship of model gene levels with tumor-infiltrating immune cell (TIIC) levels within TME. For TCGA-STAD dataset, FPKM values were transformed to transcripts per kilobase (TPM), which were suggested to be the same as those from microarray, according to previous description (14). After background adjustment and quantitative normalization of all the downloaded data, STAD was combined with GSE84437 (n=804 subjects), GSE62254 was combined with GSE15459 (n=500 subjects), and “Combat” algorithm was utilized to reduce the likelihood of batch effects from non-biological technical biases between different datasets (15). The present work excluded patients showing survival ≤30 days or those with no survival data because they might have died from lethal complications (such as bleeding, heart failure HF or intracranial infection) but not GC. Clinical variables were age, sex, T stage, clinical stage, N stage, survival status and overall survival (OS).

Furthermore, this work obtained the scRNA-seq count matrix in GSE163558 dataset. This dataset covered 10 samples, with 4 distant metastasis (DM), 3 primary tumor, 2 lymph node metastasis (LNM) and 1 corresponding para-carcinoma samples. Because our research aimed to examine TIICs levels within tumor tissues, 3 primary GC tissues (GSM5004180, GSM5004181, GSM5004182) were selected for analysis. A list of databases used with GEO accession numbers were present in **Supplementary Table S1**.

### Single-cell analysis

Quality control (QC) was completed by employing Seurat R package (16) (V4.1.1). The percentage of mitochondrial genes was computed by the “PercentageFeatureSet” function and the relationship between sequencing depth and mitochondrial gene sequences and/or total intracellular sequences was elucidated by correlation analysis. Cells with RNA count <50 and those with mitochondrial gene expression proportion >5% were eliminated from this work. The Seurat “NormalizeData” function was adopted for normalizing data, and the top 1500 genes with highly variable characteristics were confirmed by variance analysis. With false discovery rate (FDR) < 0.05, the dimensions with significant separation were filtered by principal component analysis (PCA) (17), and then the top 15 principal components (PCs) were downscaled by the t-SNE algorithm to obtain the main clusters (18). Specifically, PCs were determined by the “JackStraw” procedure. Marker genes in each cluster were accessed with log_2_ [fold change (FC)] > 0.5 and FDR < 0.05, and the top 10% of marker genes in the clusters were paved on the heat map. By using “SingleR” package (19) (V1.10.0), cells in diverse clusters were matched against the annotated reference dataset “HumanPrimaryCellAtlasData”, which was downloaded *via* the “celldex” package (V1.6.0). Each cluster was annotated based on comparison analysis as well as those cellular markers identified. For revealing cell cluster differentiation, the R package “Monocle2” (20) (V2.24.1) was applied.

### Functional enrichment and IRG-based signature establishment for prognosis prediction

For better investigating function of immune-related marker genes, these genes were subject to Gene Ontology (GO) functional annotation as well as Kyoto Encyclopedia of Genes and Genomes (KEGG) pathway analysis with “clusterProfiler” package (V4.4.4) of R. Afterwards, a prognostic immune signature was built by univariate together with multiple regression and 1000 times least absolute shrinkage and selection operator (LASSO) for predicting OS of GC patients. First, univariate regression was conducted on IRGs for determining OS-associated genes. Second, overfitting was prevented by LASSO analysis using “glmnet” R package (V4.1.4), while genes that were closely related were removed, after univariate analysis, significant genes were obtained. Thereafter, the gene contributions to predicting prognosis were assessed by multiple regression. It is worth mentioning that Akaike information criterion (AIC) values were evaluated to assist in the selection of the optimal model genes. All the GC patients of TCGA-STAD and GSE84437 were randomized as training or test group at the ratio of 1:1, then a prognostic immune-related risk score was developed.

This work later categorized altogether 402 cases from training set as high- (risk score>median) or low-risk (risk score<median) group based on median risk score, followed by Kaplan-Meier (KM) survival analysis. Similarly, test group, entire cohort, together with the merged cohort of GSE62254 and GSE15459 were classified as high- or low-risk group for KM analyses, thereafter, receiver operating characteristic (ROC) curves were plotted by the “survival” (V3.4.0) as well as “survminer” (V0.4.9) functions of R package.

### Nomogram scoring system construction and validation

Afterwards, this work applied clinical features together with immune-related risk score for developing the prediction nomograms based on results of independent prognostic analyses on the entire cohort and the merged validation set using the “rms” package. In the nomogram scoring system, a score was assigned to each variable, and score of each variable was added to calculate total score of an individual sample (21). Calibration curves for nomograms were also utilized for describing the relation between estimated 1-/3-/5-year survival events and actual observation.

### Assessment of immune status, cancer stem cell index, tumor mutation burden, and microsatellite instability between low- and high-risk subgroups

For evaluating TIIC proportion within TME, this work utilized CIBERSORT for measuring 22 TIIC proportions within heterogeneous samples from both subgroups. The associations of 22 TIIC proportions with 7 model genes and risk score were explored. Moreover, different TIIC levels between both subgroups were also analyzed for complementary purposes. In addition, different IRGs, genes related to immunogenic cell death (ICD) and necroptosis were analyzed between both subgroups from STAD-GSE84437 and external validation cohort. Generally, The Cancer Immunome Atlas (TCIA) online platform helps to comprehensively analyze immune genome (16). Tumor immunogenicity was rated at the 0-10 scale, and the score was referred to as immunophenoscore (IPS). IPS was used for predicting ICI response. Meanwhile, the online website (HTTP://tide.dfci.harvard.edu/) was adopted for calculating tumor immune dysfunction and exclusion scores. Afterwards, this work utilized the algorithm “Estimation of Stromal and Immune cells in Malignant Tumors using Expression data” (ESTIMATE) for assessing immune scores, stromal scores, and estimate scores for each GC sample (22). In addition, the relations of MSI and CSC index with risk score were also analyzed. The CSC index was calculated in the range of 0-1, with the score closer to 1 indicating the decreased cell differentiation level and enhanced CSC features. TMB scores were also calculated for GC patients from two groups in STAD-GSE84437. This work also carried out gene set variance analysis (GSVA) based on marker gene set by adopting Molecular Signatures Database (MSigDB) (c2.cp.kegg.v7.2 and c5.go.v7.2).

### Analysis of mutations and drug sensitivity

For exploring differences in therapeutic responses among chemotherapeutics between 2 groups, values of semi-inhibitory concentration (IC50) were determined for chemotherapeutics frequently adopted for CRC treatment with “pRRophetic” software.

### Validation in the external immunotherapy cohort IMvigor210

This work obtained IMvigor210 cohort at (http://research-pub.gene.com/IMvigor210CoreBiologies), a website presenting the cohort study on atezolizumab for locally advanced and metastatic uroepithelial cancer patients (23). Moreover, “arrayQualityMetrics” (V3.52.0) in R package was employed for QC of relevant microarray data, thereafter, count data were normalized by using trimmed mean of M-values. R package “limma” (V3.52.2), “voom” function was adopted for logarithm analysis (24, 25). Samples without clinical response in the IMvigor210 cohort were eliminated.

### Statistical analysis

Distributions of continuous and dichotomous variables were compared by t-test/variance and chi-square test. Separately. Survival was analyzed by log-rank test and KM statistics. Perl and R were adopted for statistical analysis, with P<0.05 indicating statistical significance.

## Results

### scRNA-seq data QC and normalization

**Figure 1:**
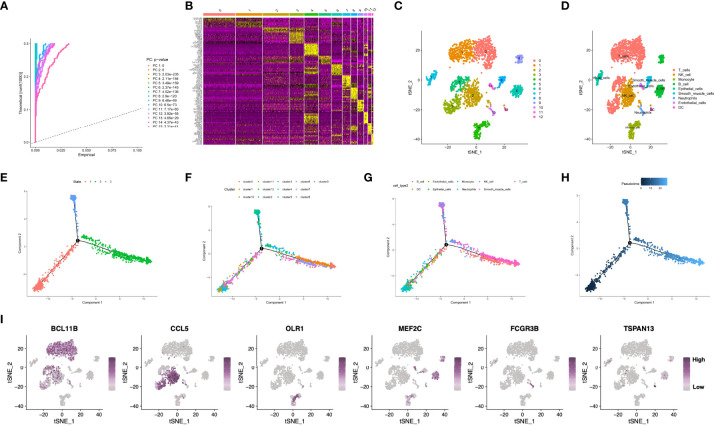
**(A)** Fifteen PCs with significant differences were identified at P<0.5. **(B, C)** All cells were clustered into thirteen clusters, and the top 10% of marker genes in each cluster are displayed on the heat map. **(D)** Nine clusters were annotated based on marker genes. **(E–H)** Pseudo-time and trajectory analysis. **(I)** The six classes of immune cells and their most prominent marker genes were shown. PCA, principal component analysis; PC, principal component; GC, gastric cancer.

The workflow of this manuscript was displayed in **Supplementary Figure S1**. The present work acquired a total of 2,157 cells from 3 GSE163558-derived primary GC samples, which passed QC (**Supplementary Figure S2A**). Sequencing depth was lowly related to mitochondrial gene sequences (R=0.01; **Supplementary Figure S2B**). Additionally, sequencing depth was positively related to total intracellular sequences (R=0.8). This work examined altogether 5,045 genes, including 1,500 and 3,545 with high and low intercellular variation, separately (**Supplementary Figure S2C**). Moreover, this work also employed principal component analysis (PCA) to reduce dimensionality of the scRNA-seq data. As a result, GC cells were not significantly segregated (**Supplementary Figure S2D**), therefore, the top 15 most significantly different PCs were chosen in later analyses (**Figure 1A**). Aggregation of cells in 13 clusters was detected by using t-distributed stochastic neighbor embedding (t-SNE) algorithm, and differential analysis detected altogether 2495 marker genes. Of them, the 10% of most significant marker genes from diverse clusters are exhibited in heatmap (**Figures 1B** **, C**). Thereafter, marker genes were utilized to annotate nine clusters, among which, clusters 0, 1, 3 and 5 were all T cells, and clusters 2, 4, 6, 7, 8, 10, 11 and 12 were correlated with natural killer (NK) cells, monocytes, B cells, epithelial cells, smooth muscle cells, neutrophils, endothelial cells, as well as dendritic cells (DCs), respectively (**Figure 1D**). Pseudo-time and trajectory analysis indicated that clusters 0, 3, 6, 9, 11 were distributed in subset I; whereas clusters 1, 4, 7, 12 were in subset II; while clusters 2, 5, 8, 10 were distributed in subset ; and the cell types were also shown (**Figures 1E–H**). The 6 classes of immune cells and their most prominent marker genes are presented in **Figure 1I**. Afterwards, a total of 1424 marker genes of 6 classes of immune cells were selected in later analyses (**Supplementary Table S2**).

### Gene enrichment analysis and immune-related model construction

**Figure 2:**
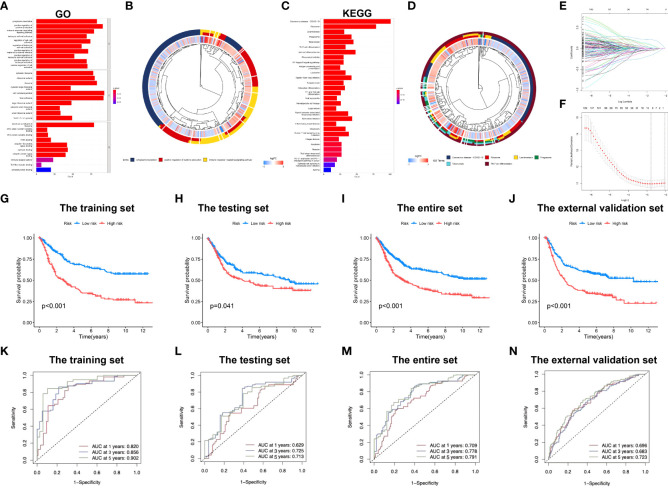
Development of the immune-related signature in the training set. **(A)** GO functional enrichment analysis of immune-related genes. **(B)** Concentric circle diagram of the GO analysis. **(C)** KEGG pathway analysis of immune-related genes. **(D)** Concentric circle diagram of the KEGG analysis. **(E)** LASSO coefficient curves are shown. **(F)** Selection of tuning parameters (lambda) in the LASSO model by tenfold cross-validation according to the minimum criterion of OS is displayed; the lower x-axis indicates the log (lambda) and the upper x-axis the average number of OS-gene. The y-axis represents the partial likelihood bias error. The red dots denote the average partial likelihood deviation for each model given the lambda, and the vertical bars indicate the upper and lower values of the partial likelihood deviation error. Kaplan-Meier survival curves of patients in low-risk group and high-risk group of the training set **(G)**, the testing set **(H)**, the entire set **(I)**, and the external validation cohort **(J)** are presented. **(K–N)** indicate survival-dependent ROC curves validation at 1-year, 3-year and 5-year of prognostic value of the signature in the four sets (the training set, the testing set, the entire set, and the external validation cohort, respectively). GO, Gene Ontology; KEGG, Kyoto encyclopedia of genes and genomes; LASSO, least absolute shrinkage and selection operator; OS, overall survival; ROC, receiver operating characteristic curves.

Enrichment analysis of marker genes for immune cells obtained from the profiling of scRNA-seq data was conducted, and our results demonstrated that these genes were enriched into a variety of immune-related GO entries, such as “immune response regulating signaling pathway”, “positive leukocyte activation regulation”, “positive cytokine production regulation”, “T-cell activation regulation” and “cell activation involved in immune response” (**Figures 2A** **, B**; **Supplementary Table S3**). In terms of KEGG pathways, “T cell receptor pathway”, “Toll-like receptor pathway”, “B cell receptor pathway” and “TNF pathway” were featured based on our findings. Moreover, several cancer-associated pathways, including “MAPK pathway” and “transcriptional dysregulation within cancer” were also shown (**Figures 2C** **, D**; **Supplementary Table S4**).

Subsequently, the immune-related signature was built based on the identified marker genes. Patients were split in to training and testing sets through R language “caret package”. After univariate regression analysis, 126 genes associated with OS were screened, and finally 7 were identified as our model genes by LASSO and multiple regression based on the minimum partial likelihood deviance (**Figures 2E** **, F**). Thereafter, risk score was evaluated as follows, [SLAMF7 level* (-0.2529)] + [DUSP level* (0.2349)] + [APLP2 level* (0.2582)] + [FLOT1 level* (0.3437)] + [EEF2 level* (-0.2562)] + [TRIM25 level* (-0.4191)] + [UGCG level* (0.2501)]. All cases were divided as low- or high-risk group according to median risk score value.

**Figure 3:**
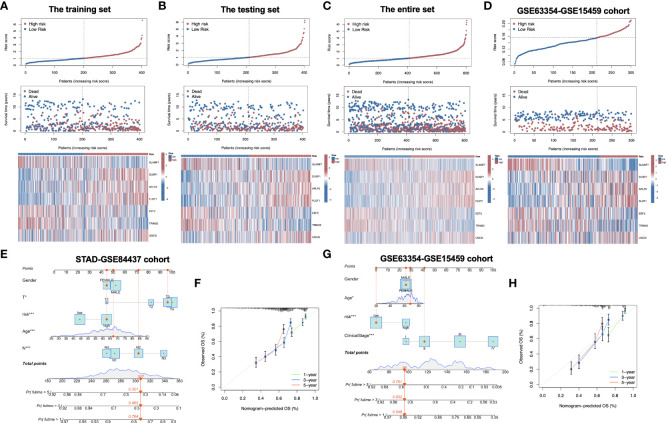
Distribution of risk score, overall survival, gene expression in the training set **(A)**, the testing set **(B)**, the entire set **(C)** and the external validation cohort **(D)**. Distribution of risk score, overall survival, and heatmap of the expression of eight signature genes in low-risk and high-risk groups is presented in the figure from top to bottom. **(E)** Nomogram for predicting the 1-year, 3-year and 5-year OS of GC patients in the entire set. **(F)** Calibration curves of the nomogram for predicting of 1-year, 3-year and 5-year OS in the entire set. **(G)** Nomogram for predicting the 1-year, 3-year and 5-year OS of GC patients in the external validation cohort. **(H)** Calibration curves of the nomogram for predicting of 1-year, 3-year and 5-year OS in the external validation cohort. OS, overall survival; GC, gastric cancer. *P<0.05; ***P<0.001.

**Figure 2G** compares differences in survival of both risk subgroups from the training set (P<0.001) in STAD-GSE84437. The results were subsequently confirmed with test set (P<0.05; **Figure 2H**) as well as the combined set (P<0.001; **Figure 2I**). Besides, survival in the GSE62254 and GSE15459 external validation cohort was similar (P<0.001; **Figure 2J**). Furthermore, this work determined the areas under the curves (AUCs) for 1-year OS to be 0.820, 0.629, 0.709 and 0.696; 0.856, 0.725, 0.778 and 0.683 for 3-year OS; and 0.902, 0.714, 0.791 and 0.723 for 5-year OS for training, test, entire and external validation sets, respectively. These results suggested that our gene signature had moderate potential for monitoring survival (**Figures 2K** **–N**). Distributions of survival status, risk scores, and gene feature expression of training (**Figure 3A**), test (**Figure 3B**), entire (**Figure 3C**) and external validation sets (**Figure 3D**) were obtained. Apparently, similar distributions were observed, which supported that our constructed risk score model was of great prediction power.

To explore whether the constructed IRG model was significant for the independent prognosis prediction, this work carried out univariate as well as multiple analysis. As a result, risk score possibly independently predicted patient prognosis in STAD-GSE84437 dataset (hazard ratio HR:1.318, 95% confidence intervals CIs: 1.188−1.463, P<0.001) and external validation set (HR: 2.272, 95% CIs: 1.596−3.235, P<0.001) (**Tables 1**, **2**, respectively). Afterwards, nomograms were constructed for achieving more accurate personalized prediction for GC patients in the entire set (**Figure 3E**) and the external validation set (**Figure 3G**). The former consisted of age, risk score, T stage, and N stage, while the latter included age, risk score and clinical stage. As revealed by calibration curves, the model-predicted values were consistent with real observed OS, which indicated the excellent statistical power of the predicted survival for both sets (**Figures 3F** **, H**). The dotted line indicates the perfect nomogram, whereas the solid one represents the current nomogram.

**Table 1:** Univariate and Multivariate Cox regression analyses of clinicopathologic characteristics associated with overall survival in the TCGA-GSE84437 cohort.

Variable	Univariate analysis		Multivariate analysis	
	HR (95% CI)	*P -* value	HR (95% CI)	*P -* value
Age (>60/≤60)	1.025 (1.016-1.036)	<0.001^a^	1.029 (1.019-1.040)	<0.001^a^
Gender (male/female)	1.244 (0.986-1.569)	0.065	–	–
T stage (T1/T2/T3/T4)	1.256 (1.093-1.442)	<0.001^a^	1.190 (1.025-1.381)	<0.001^a^
N stage (N0/N1/N2/N3)	1.549 (1.383-1.735)	<0.001^a^	1.443 (1.282-1.623)	<0.001^a^
Risk score	1.415 (1.279-1.566)	<0.001^a^	1.319 (1.188-1.463)	<0.001^a^

a Statistically significant. TCGA, The Cancer Genome Atlas; HR, Hazard ratio; CI, confidence interval.

**Table 2:** Univariate and multivariate Cox regression analyses of clinicopathologic characteristics associated with overall survival in the GSE63354-GSE85459 cohort.

Variable	Univariate analysis		Multivariate analysis	
	HR (95% CI)	*P -* value	HR (95% CI)	*P -* value
Age (>60/≤60)	1.007 (0.996-1.018)	0.013^a^	1.013 (1.003-1.025)	0.013^a^
Gender (male/female)	1.066 (0.818-1.390)	0.637	–	–
Clinical stage (I/II/III/IV)	2.458 (2.101-2.876)	<0.001^a^	2.431 (2.076-2.846)	<0.001^a^
Risk score	2.766 (1.947-3.929)	<0.001^a^	2.272 (1.596-3.235)	<0.001^a^

a Statistically significant. HR, Hazard ratio; CI, confidence interval.

### Evaluation of the TIME between low- and high-risk subgroups

**Figure 4:**
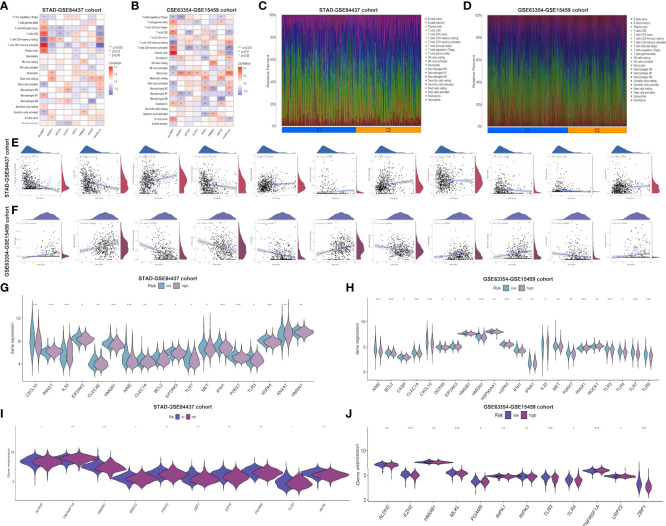
Evaluation of the different immune status between the low- and high-risk groups in both cohorts. Correlations between the seven model genes as well as the risk score and the abundance of immune cells in STAD-GSE84437 **(A)** and the external validation cohort **(B)**. The proportions of various immune cells in samples of STAD-GSE84437 **(C)** and the external validation cohort **(D)** were presented. **(E, F)** Relationships between risk score and immune cell types in STAD-GSE84437 and the external validation cohort, respectively. **(G, H)** Differential expression of ICD-related genes between high- and low-risk groups in STAD-GSE84437 and the external validation cohort, respectively. **(I, J)** Differential expression of necroptosis−related genes between high- and low-risk groups in STAD-GSE84437 and the external validation cohort, respectively. STAD, stomach adenocarcinoma; ICD, immunogenic cell death. *P<0.05; **P<0.01; ***P<0.001.

This work employed CIBERSORT algorithm for estimating correlation of immune-related risk score with TIIC levels. As demonstrated in **Figure 4A**, the risk score showed positive relation to M2 macrophages, resting memory CD4^+^ T cells, mast cells, and naive B cells, whereas negative relation to activated memory CD4^+^ T cells, follicular helper T cells, CD8^+^ T cell, M1 macrophages, plasma cells and memory B cells within STAD-GSE84437 dataset. For low- and high-risk subgroups of external validation set, the similar pattern of immune infiltration was observed. M2 macrophages, monocytes, mast cells, resting memory CD4^+^ T cells and neutrophils were positively related to the risk score, while activated memory CD4^+^ T cells, resting NK cells, CD8^+^ T cells, naive B cells, M1 macrophages, and plasma cells showed negative relation to risk score (**Figure 4B**). Besides, relations between 7 model genes and different TIIC abundances were displayed together. **Figures 4C** **, D** presents TIIC abundances within samples of two combined datasets. Relationships between risk score and immune cell types in STAD-GSE84437 and the external validation cohort were also shown (**Figures 4E, F**). Previous studies have demonstrated that ICD modulator genes and necroptosis−related genes have critical effects on the anticancer immune responses in the host. Afterwards, differential analysis on ICD-related and necroptosis−related genes was conducted on low- and high-risk subgroups of two combined datasets. This work obtained the above genes from previously published documents (26, 27) (**Supplementary Tables S5, S6**). It was found that in the either set, most of the ICD-related genes, including HMGB1, CXCL10, AIM2 and HSPA4 showed up-regulation among low-risk patients, whereas PANX1, IL33, ROCK1 and ANXA1 showed over-expression among high-risk patients (**Figures 4G** **, H**). Similarly, most of the necroptosis−related genes, including EZH2, ZBP1, PGAM5 and ALDH2, were found to be highly expressed among low-risk patients relative to high-risk counterparts from two merged datasets (**Figures 4I, J**).

**Figure 5:**
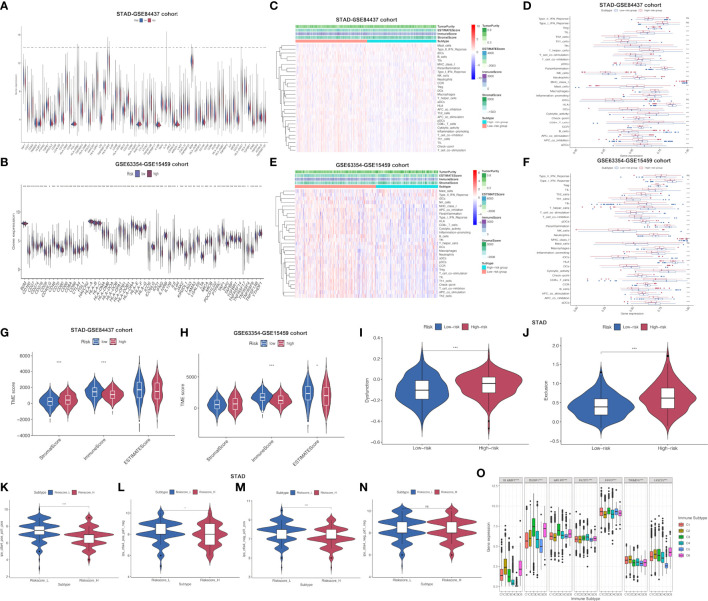
Assessment of distinct immune landscapes between high-and low-risk groups in both cohorts. **(A, B)** Differential expression of immune−related genes between high- and low-risk groups in STAD-GSE84437 and the external validation cohort, respectively. **(C, D)** Immune-related functions differ between high- and low-risk groups for STAD-GSE84437. **(E, F)** Immune-related functions differ between high- and low-risk groups for the external validation cohort. **(G, H)** Differences in immune score, stromal score, and ESTIMATE score between low- and high-risk groups in STAD-GSE84437 and the external validation cohort, respectively. **(I)** Different T cell dysfunction scores between high- and low-risk groups in STAD-GSE84437. **(J)** Different T cell exclusion scores between high- and low-risk groups in STAD-GSE84437. **(K–N)** Analysis of potential differential responses to immune checkpoint inhibitors therapy in low- and high-risk groups of patients in TCGA-STAD using TCIA data. **(O)** Association of the expression of model genes with immune infiltrate subtypes across all the cancer types in TCGA. STAD, stomach adenocarcinoma; ESTIMATE, estimation of Stromal and Immune cells in Malignant Tumors using Expression data; TCIA, The Cancer Immunome Atlas. *P<0.05; **P<0.01; ***P<0.001.

Furthermore, common immune gene levels in low- and high-risk groups were also analyzed. Several genes intimately involved in immunosuppression, such as TGFBR1, TGFB1 and VTCN1, showed high expression levels among high-risk patients of STAD-GSE84437 (**Figure 5A**). By contrast, several immunostimulators (CD27, CD48, TNFRSF13B, TNFRSF14 and TNFRSF25) were closely associated with the low-risk patients. Nonetheless, PD-1, CTLA-4, and PD-L1, the three vital immune checkpoint-associated genes, showed significant over-expression among low-risk patients. As for external validation cohort, TNFRSF25, ICOS, CD27, CD28, CD48 and TNFRSF9, which have been demonstrated with immunostimulatory effects, showed high expression levels among low-risk patients relative to high-risk cases (**Figure 5B**). Meanwhile, PD-L1, CTLA4 and PD-L2 also demonstrated a stronger association with low-risk patients. Various immune functions between low- and high-risk cases from both cohorts were compared, which indicated that most IRG functions, like “check point”, “chemokine receptors (CCR)”, “inflammation-promoting”, and “human leukocyte antigen (HLA)”, showed higher enrichment levels among low-risk cases, which further reflected the greater number and complexity of immune components among low-risk patients (**Figures 5C** **–F**). Afterwards, high-risk patients had remarkably increased stromal scores compared with low-risk patients from the STAD-GSE84437 dataset (**Figure 5G**). Additionally, low-risk patients had markedly increased immune scores relative to high-risk patients from both cohorts (**Figures 5G** **, H**). The ESTIMATE score was not significantly different between low- and high-risk patients in STAD-GSE94437 (**Figure 5G**), but low-risk patients had increased scores compared with high-risk patients from the external validation dataset (**Figure 5H**). Moreover, high-risk patients were associated with the increased T-cell exclusion scores as well as T cell disfunction scores in comparison with low-risk patients (**Figures 5I** **, J**). Additionally, TCIA was employed to anticipate the sensitivity of patients in TCGA-STAD to immunotherapy. It was found that low-risk patients had markedly elevated expression of ips-ctla4-pos-pd1-pos (**Figure 5K**), ips-ctla4-pos-pd1-neg (**Figure 5L**) and ips-ctla4-neg-pd1-pos (**Figure 5M**) compared with high-risk patients. Nevertheless, ips-ctla4-neg-pd1-neg expression was not significantly different between two subgroups (**Figure 5N**). Low-risk patients had increased IPS compared with high-risk patients, suggesting the higher ICI sensitivity of low-risk patients.

To obtain an understanding of how each model gene was related to the immune component, associations of model genes with TIICs levels within human cancers were analyzed. To be specific, 6 TIICs levels were analyzed within human tumors, corresponding to a range from tumor progression promotion to inhibition (28). The immune infiltration was classified as 6 subtypes, including C1 (wound healing), C2 (INF-r dominance), C3 (inflammation), C4 (lymphocyte depletion), C5 (immune quiet) as well as C6 (TGFβ dominance) (**Supplementary Table S7**). Immune infiltration levels were examined based on pan-caner data from TCGA, which were later associated with our model gene levels. As revealed in previous research (28), patients classified as C3 and C5 subtypes were associated with remarkably superior OS to those of the remaining subtypes (P<0.0001), typically, C4 and C6 subtypes indicated the poorest prognosis. SLAMF7, TRIM25 and UGCG up-regulation was related to C2 and C6 subtypes, indicating the tumor promoting effect of the above genes, because cases of the above subtypes showed poor survival with TGFβ enrichment and enhanced proliferation. FLOT1 had higher expression in C4 subtype, and it predicted the dismal survival, which suggested the tumor promoting effect. On the contrary, DUSP1, APLP2 and EEF2 up-regulation was related to C3 subtype rather than additional subtypes, which suggested that up-regulated gene levels were related to favorable immune components, indicating the tumor suppressor effects of the above genes (**Figure 5O**). Moreover, **Supplementary Figure S3** presents the association of model genes in STAD-GSE84437 and the external validation set.

### Comprehensive analysis on MSI, TMB, enrichment functions and drug sensitivity

**Figure 6:**
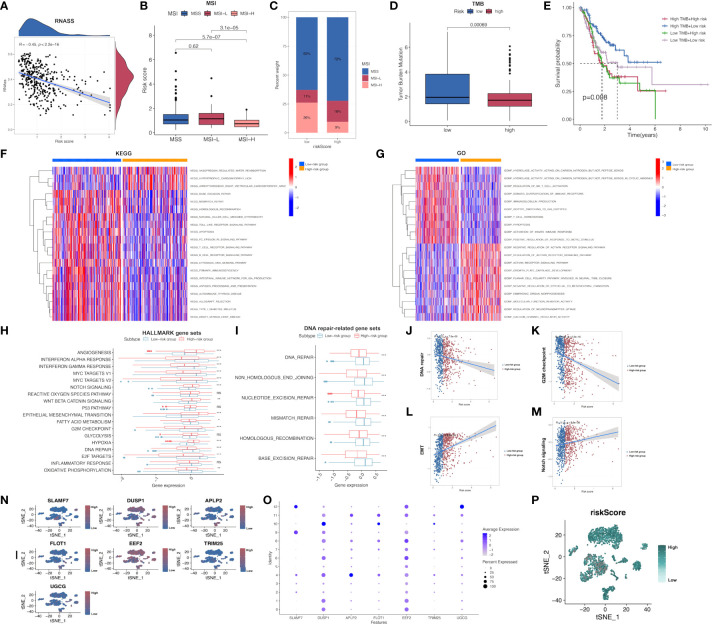
Comprehensive comparison of the differences between high- and low-risk groups screened by our immune-related model. **(A)** Relationships between risk score and CSC index. **(B, C)** Relationships between risk score and MSI. **(D)** TMB in low- and high-risk groups in STAD-GSE84437. **(E)** Relationships between TMB and prognosis of GC patients in STAD-GSE84437. **(F)** Differential enrichment of KEGG pathways between low- and high-risk groups in STAD-GSE84437. **(G)** Differential enrichment of GO annotations between low- and high-risk groups in STAD-GSE84437. **(H)** Differential enrichment of Hallmark pathways between low- and high-risk groups in STAD-GSE84437. **(I)** Differential enrichment of DNA repair-related pathways between low- and high-risk groups in STAD-GSE84437. **(J–M)** Spearman correlation analysis between risk scores and DNA repair, G2M checkpoint, EMT and Notch signaling, respectively. **(N, O)** The expression of seven model genes in the primary GC tissues in scRNA-seq dataset GSE163558. **(P)** The distribution of risk scores of different cells in primary GC tissues in GSE163558. CSC, cancer stem cell; TMB, tumor mutation burden; MSI, microsatellite instability; GO, Gene Ontology; KEGG, Kyoto encyclopedia of genes and genomes; GC, gastric cancer; STAD, stomach adenocarcinoma; EMT, epithelial-mesenchymal transition; scRNA-seq, single-cell RNA sequencing. *P<0.05; **P<0.01; ***P<0.001.

In addition, the possible association of risk score with CSC index was evaluated. As revealed in Figure, risk score showed negative relation to CSC index (R=-0.45, P<0.001; **Figure 6A**), demonstrating that GC cells with higher risk score had pronounced stem cell features whereas decreased cell differentiation. Subsequently, the relationship between MSI and risk score was investigated. According to correlation analysis, low risk score showed significant relation with high-frequency MSI (MSI-H) status, whereas high risk score showed relation with microsatellite stability (MSS) status (**Figures 6B** **, C**). Cumulative evidence suggested that patients with high TMB could possibly gain benefits from immunotherapy because of the high neoantigen number. Mutation data obtained from TCGA-STAD set were also analyzed, which demonstrated that low-risk patients had increased TMB compared with high-risk patients (P<0.001; **Figure 6D**). This work further investigated the relationship between TMB and the prognosis of both risk subgroups in STAD, as a result, cases with low-risk and high TMB showed the longest OS, followed by patients with low-risk and low TMB, whereas patients with high-risk and high/low TMB displayed the poorest prognosis (**Figure 6E**).

Afterwards, GSVA was performed to investigate the different GO, KEGG and Hallmark gene functions between two risk subgroups from STAD-GSE84437. Our results showed that both risk subgroups showed differential enrichment into multiple KEGG pathways, including “Mismatch repair”, “Apoptosis” and “homologous recombination” (**Figure 6F** and **Supplementary Table S8**). Meanwhile, some immune-related pathways, including “B cell receptor pathway”, “T cell receptor pathway”, “Natural killer cell mediate cytotoxicity” and “Toll like receptor pathway” were also featured in our enrichment list, which showed higher enrichment levels among low-risk patients. GO annotation highlighted the higher enrichment levels of several items such as “Regulation of activin receptor pathway”, “Growth plate cartilage development” and “Molecular function inhibitor activity” among high-risk patients (**Figure 6G** and **Supplementary Table S9**). In addition, GO analysis also suggested that “NKT cell activation regulation”, “Immunoglobulin production”, “T-cell homeostasis” and “Activation of innate immune response” were functions more significantly associated with low-risk patients in comparison with high-risk patients. Differences in GO and KEGG analyses between both risk subgroups from the external validation set are presented in the **Supplementary Figures S4**, **S5** and **Supplementary Tables S10, S11**, respectively. Afterwards, the different Hallmark gene functions were compared between two risk subgroups. Pathways associated with tumorigenesis, like “Hypoxia”, “Angiogenesis”, and “Epithelial-mesenchymal transition (EMT)” were more closely related to high-risk cases, while “G2M checkpoint”, “DNA repair”, “MYC target V1 and V2”, “Interferon α and γ response” were significantly enriched among low-risk cases (**Figure 6H**). Considering that DNA damage-related pathways were associated with TMB, the differential enrichment of DNA damage-related pathways between two risk subgroups was compared. As a result, “Mismatch repair”, “Base excision repair”, “Non homologous end joining”, “Nucleotide excision repair” and “Homologous recombination” were highly enriched into low-risk subgroup (**Figure 6I**), conforming to our previous TMB analysis. Additionally, four representative pathways, namely DNA repair (R=-0.2; P<0.001), G2M checkpoint (R=-0.32; P<0.001), Notch signaling pathway (R=0.19; P<0.001) and EMT (R=0.4; P<0.001) were selected, and the Spearman correlation coefficients with risk score were shown (**Figures 6J–M**). Furthermore, the expression of 7 model genes in the primary GC tissues from the scRNA-seq dataset GSE163558 is displayed in **Figures 6N, O**. Meanwhile, the risk scores of different cells in primary GC tissues of GSE163558 were calculated by calculating risk scores, as presented in **Figure 6P**, with darker colors representing the higher risk.

**Figure 7:**
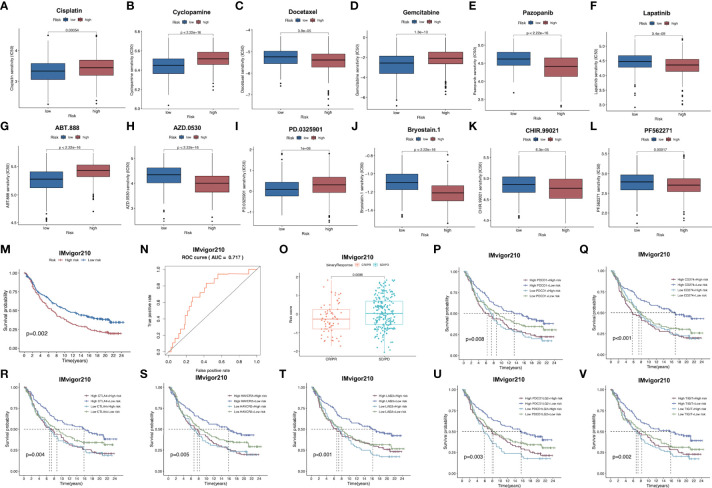
**(A–L)** Corrections between risk score and chemotherapeutic sensitivity. **(M)** Kaplan-Meier survival curves of patients in low-risk group and high-risk group of the IMvigor210 cohort. **(N)** Relationships between risk scores and response to immunotherapy in the IMvigor210 cohort **(O)** Differences in risk scores between patients with SD/PD and CR/PR in IMvigor210 cohort.. **(P–V)** Effect of expression of 5 key immune checkpoints (PD-1, PD-L1, PD-L2, CTLA4, TIGIT, LAG3 and HAVCR2, respectively on OS of patients in low- and high-risk groups in IMvigor210 cohort. OS, overall survival; PR, partial response; CR, complete response; PD, progressive disease; SD, stable disease.

Subsequently, patient sensitivity to commonly adopted chemotherapeutics and targeted drugs among low-and high-risk patients was analyzed. Intriguingly, high-risk patients were associated with decreased IC50 of docetaxel as well as a number of targeted drugs, such as lapatinib, pazopanib, AZD.0530, Bryostin.1, CHIR.99021, PF.562271, while low-risk cases had remarkably decreased IC50 of gemcitabine, cisplatin, cyclosporine, ABT.888 and PD.0325901. Collectively, these results indicated that the risk model developed by our study was related to drug sensitivity (**Figures 7A** **–L**; **Supplementary Figure S6**).

### Immune-related signature validation for predicting immunotherapeutic response in the IMvigor210 database

For exploring the role of our constructed immune signature in predicting immunotherapy benefits, this work explored the independent data from the publicly available IMvigor210 study. The formula of our model allowed to classify cases in IMvigor210 database as two low- or high-risk subgroup. Notably, low-risk cases showed remarkably prolonged OS compared to high-risk cases (**Figure 7M**). Furthermore, AUC was 0.717 for 1-year OS, indicating that the gene signature was potent in monitoring survival (**Figure 7N**). In addition, patients with partial response (PR) and complete response (CR) in IMvigor210 database were associated with remarkably increased risk scores relative to those with progressive disease (PD) and stable disease (SD), which implied that immunotherapy was more beneficial for low-risk patients, conforming to our prior analysis (**Figure 7O**). Furthermore, relationships between 7 key immune checkpoints levels (PD-1, PD-L1, PD-L2, CTLA4, TIGIT, LAG3 and HAVCR2) and the prognosis of patients from both risk subgroups are presented in **Figures 7P** **–V**. As a result, up-regulation of the above 7 genes combined with low risk scores predicted the remarkably prolonged OS.

## Discussion

GC was identified as the second most common cause of cancer deaths worldwide (29). The main treatment modalities available to patients include surgical resection, perioperative chemotherapy or chemoradiotherapy, adjuvant chemotherapy or chemoradiotherapy, but the benefits from these are limited due to the heterogeneous nature of the disease. In the last decade, immune checkpoint blockade has emerged as an attractive therapeutic strategy in a variety of malignancies, including GC (30). The tumor immune microenvironment shows genetic and transcriptional diversity and plays important roles in tumor progression, metastasis, and treatment resistance (31). Therefore, exploring the immune microenvironment of GC from various perspectives and mining novel immune-related genetic models could contribute to our further insight into the immune landscape of GC and predict the immunotherapeutic response of patients. In addition, single-cell sequencing technologies are rapidly evolving and have the ability to finely characterize the vast heterogeneity within tumors (32). Combining single cell sequencing data with transcriptomic data to analyze the immune microenvironment status of GC represents a novel and reliable approach.

To begin with, we analyzed scRNA-seq data of GC to obtain the immune cell types infiltrated by tumor tissue and obtained their marker genes. Subsequently, we performed functional enrichment analysis of the marker genes and demonstrated their enrichment in multiple immune-related pathways. Afterwards, a 7-immune gene-based risk scoring model was constructed and demonstrated. Remarkably, it can distinguish between high- and low-risk individuals, and the prognosis was estimated with high accuracy. GC patients in the low-risk group were proven to experience longer OS than those in the high-risk group in the training set, the testing set, the entire set and the external validation cohort. The nomograms were developed to provide a more comprehensive view of the predictive capability of our signature by incorporating clinical characteristics.

Subsequently, we concentrated on uncovering the different immune infiltration profiles of high- and low-risk patients screened by our constructed immune-related signature. The results indicated that a high proportion of M2 macrophages, mast cells, resting memory CD4^+^ T cells and naive B cells were observed in high-risk patients compared to low-risk patients in STAD-GSE84437, while activated memory CD4^+^ T cells, CD8^+^ T cell, M1 macrophages, memory B cells and plasma cells exhibited the opposite trend. Tumor-associated macrophages (TAMs) of the M2 phenotype are known to promote tumor proliferation and to be associated with a poor prognosis in numerous cancers (33). Pervious research (34) has demonstrated that M2 macrophages promoted the migration and invasion of GC cells *via* EMT. The researchers also illustrated that GC-derived mesenchymal stromal cells could contribute to M2 macrophage polarization through considerable secretion of IL-6 and IL-8. In our study, high M2 macrophage infiltration and high EMT status were observed simultaneously in the high-risk group, which is in agreement with previous findings. Besides, M1 macrophages have been shown to be related to better survival in GC patients (35). Sammarco et al. (36) have demonstrated that mast cell density is increased in GC and there is a correlation with angiogenesis, the number of metastatic lymph nodes and the survival of these patients. Intriguingly, patients in the high-risk group in our research not only had a higher level of mast cell infiltration, but also had a greater angiogenic state than patients in the low-risk group, which is again consistent with the published literature. In terms of T cells, Ning et al. (37) have indicated that high resting memory CD4^+^ T cells was significantly associated with poorer OS in GC while high abundance of activated memory CD4^+^ T cells was associated with better survival, which corroborates our analysis. It has been well documented that CD8^+^ T cells exert superior antitumor effects with strong retention and cytotoxicity (38, 39). Higher CD8^+^ T cells infiltration in patients in the low-risk group might be a critical factor in their better prognosis as compared to the high-risk patients. Another immune cell that was highly expressed in the high-risk group, naive B cells, has been described to have a higher degree of infiltration in tertiary lymphoid structures (TLSs)-poor GC tissues than in TLSs-rich ones (40). TLSs, which consist of B cells, T cells, follicular dendritic cells and high endothelial venules, have recently been found to be associated with effective antitumor immune responses in patients with cancer. We therefore hypothesize that there may be a link between infiltration of highly naive B cells and lower immunotherapy response in GC patients. Moreover, high infiltration of memory B cell and plasma cell have been described to indicate longer OS in GC (41), which that emphasizes their role as protective factors. In validation of the external merged cohort, two additional immune cells, monocytes and neutrophils, were revealed to be associated with higher risk scores. Wang et al. (42) have illustrated that tumor-activated neutrophils in GC foster immune suppression and disease progression through GM-CSF-PD-L1 pathway, which contributed to our understanding of the potential association of immunosuppressive status with higher neutrophil infiltration in the high-risk group of patients in the external validation cohort.

To further investigate the distinct immune profiles between high- and low risk-groups, we compared the expression of various immune-related genes, immune scores as well as immune-related functions. The results indicated that genes with immunostimulatory effects were more significantly expressed in the low-risk group than in the high-risk group, while most immune-related functions, such as cytolytic activity and HLA-related functions, were likewise more pronounced in the low-risk group, thus revealing a more active immune status in the low-risk group. Meanwhile, the higher T cell exclusion scores and T cell disfunction scores appearing in the high-risk group in STAD-GSE84437 implied aberrant T cell function and a state of immunosuppression in the high-risk group compared to the low-risk group. The expression of several immune checkpoint genes, such as CTLA4 and PD-L1, were significantly elevated in the low-risk group, revealing that the low-risk group may be more likely to benefit from immunotherapy. The next TCIA and TMB analysis also confirms the above deduction. To further investigate the potential reasons for the higher response to immunotherapy in the low-risk group, we examined the differential status of ICD and necroptosis between the two groups and demonstrated that the majority of these two-cell death-related genes were more highly expressed in the low-risk group. ICD has been defined by the emission of a range of immunostimulatory damage-associated molecular patterns (DAMPs) and then stimulate an immune response against dead-cell antigens, in particular when they derive from cancer cells (43). Necroptosis has also been illustrated to accelerate cancer cell death or enhance the sensitivity of tumor cells to anti-cancer treatment (44). Increased cell death of these two types in the low-risk group may account for their greater susceptibility to benefit from immunotherapy. Due to the lack of publicly available immunotherapy cohorts related to GC, we performed external immunotherapy response validation by selecting IMvigor 210 dataset. The results also confirmed the ability of our constructed signature to accurately predict the response of patients receiving immunotherapy.

In the ensuing comprehensive comparison, we found that the risk score was lower in the MSI-H group compared to the MSI-L and MSS groups in STAD-GSE84437. MSI-H GC has a better prognosis compared with the MSS counterpart, which is accompanied by a decreased risk of lymph node metastasis, tumor invasion as well as mortality (45). Moreover, TMB-related pathways including mismatch repair, base excision repair and DNA repair that have been identified in the previous research (46) were strongly enriched in the low-risk group, which partly explains the higher TMB in the low-risk group. Cho et al. (47) have pointed out that GC with high TMB was most highly concentrated in MSI-H groups, which is certainly in line with the results of our analysis. Moreover, angiogenesis (48), Notch pathway (49), hypoxia (50) and EMT (51) that featured in high-risk group could provide insight into the potential causes of their worse prognosis. The scRNA-seq data were further utilized to validate the expression levels of signature genes at the cellular level and to demonstrate a high and low distribution of risk scores. However, the detailed associations still require further exploration. After sensitivity analysis of multiple drugs, we hypothesized that the low-risk group would be better suited to receive immunotherapy and chemotherapy, while the high-risk group appears to benefit from treatment with various targeted agents.

However, this study has several limitations. First, all analyses were based exclusively on data from public databases, and all samples utilized in our study were retrospective. Therefore, inherent case-selection bias may have impacted the results. Large prospective studies and additional *in vivo* and *in vitro* experimental studies are needed to confirm our findings. Furthermore, data on a few important clinical characters, such as surgery and chemoradiotherapy, were not available for analysis in most data sets, and we had to remove missing clinical data from some datasets due to the merging of datasets, which may have influenced the result of our research.

## Conclusion

The present study identifies marker genes for immune cells associated with GC based on scRNA-seq and constructs a signature that can accurately predict OS, tumor microenvironment score, immune infiltration status, and response to immunotherapy in GC patients from independent databases. A nomogram based on a combination of model features and clinicopathological variables provided an intuitive and accurate method for predicting OS of patients. In summary, this study constructs a signature to predict clinical outcomes and potential drug treatment including immunotherapy response in patients starting from the single cell level, thus offering novel ideas to guide personalized immunotherapeutic strategies for GC patients.

## Data availability statement

The datasets presented in this study can be found in online repositories. The names of the repository/repositories and accession number(s) can be found in the article/**Supplementary Material**.

## Author contributions

BH created the idea for the paper. BH and YM performed the selection of literature, drafted the manuscript, and prepared the figures. CQ, B-YW and D-RX revised the manuscript. All authors read and approved the final manuscript.

## Acknowledgments

The authors would like to thank the GEO and TCGA databases for the availability of the data.

## Conflict of interest

The authors declare that the research was conducted in the absence of any commercial or financial relationships that could be construed as a potential conflict of interest.

## Publisher’s note

All claims expressed in this article are solely those of the authors and do not necessarily represent those of their affiliated organizations, or those of the publisher, the editors and the reviewers. Any product that may be evaluated in this article, or claim that may be made by its manufacturer, is not guaranteed or endorsed by the publisher.

## Supplementary material

Supplementary Figure 1 Workflow chart of the present study

Supplementary Figure 2 Quality control and normalization of scRNA-seq data, dimensionality reduction and cell trajectory analysis. **(A)** After quality control and normalization, a total of 2057 cells were screened for further analysis. **(B)** Correlation analysis between sequencing depth and mitochondrial gene sequences as well as total intracellular sequences. **(C)** A total of 5,045 genes were analyzed, of which 3,545 genes had small intercellular variation and 1,500 genes had large variation. **(D)** PCA based on scRNA-seq data.

Supplementary Figure 3
**(A)** Spearman correlation analysis of model genes in STAD-GSE84437. **(B)** Spearman correlation analysis of model genes in the external validation cohort.

Supplementary Figure 4 Differential enrichment of GO annotations between low- and high-risk groups in the external validation cohort.

Supplementary Figure 5 Differential enrichment of KEGG pathways between low- and high-risk groups in the external validation cohort.

Supplementary Figure 6 Differences in drug sensitivity between low- and high-risk groups in STAD-GSE84437.
