# Supplementary figures and images for: Combining single-cell sequencing data to construct a prognostic signature to predict survival, immune microenvironment, and immunotherapy response in gastric cancer patients
Source: Front Immunol. 2022 Oct 10;13:1018413. doi: 10.3389/fimmu.2022.1018413 (PMC9589350; doi:10.3389/fimmu.2022.1018413)

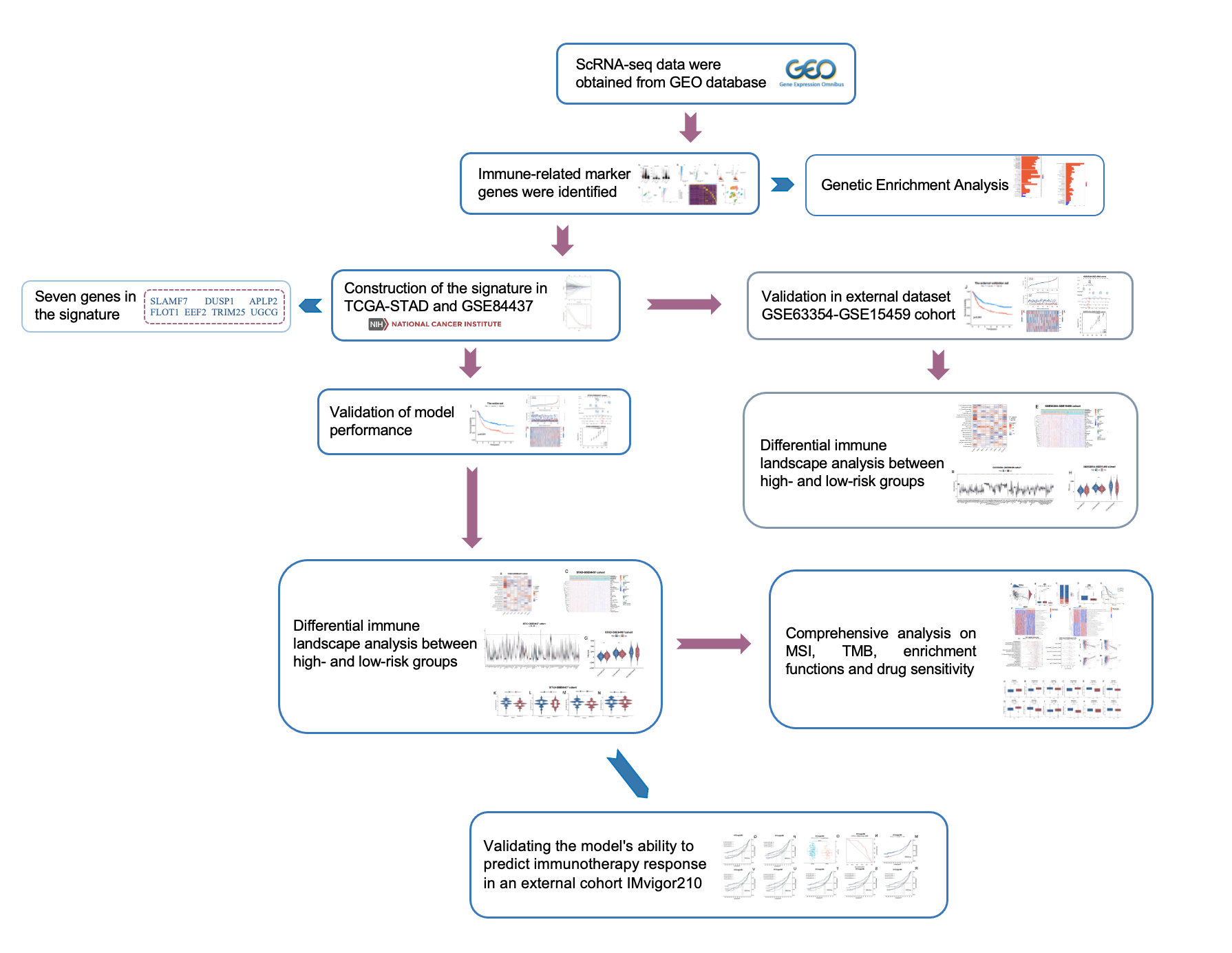

Supplement: Supplementary Figure 1 — Workflow chart of the present study [file Image_1.tiff]

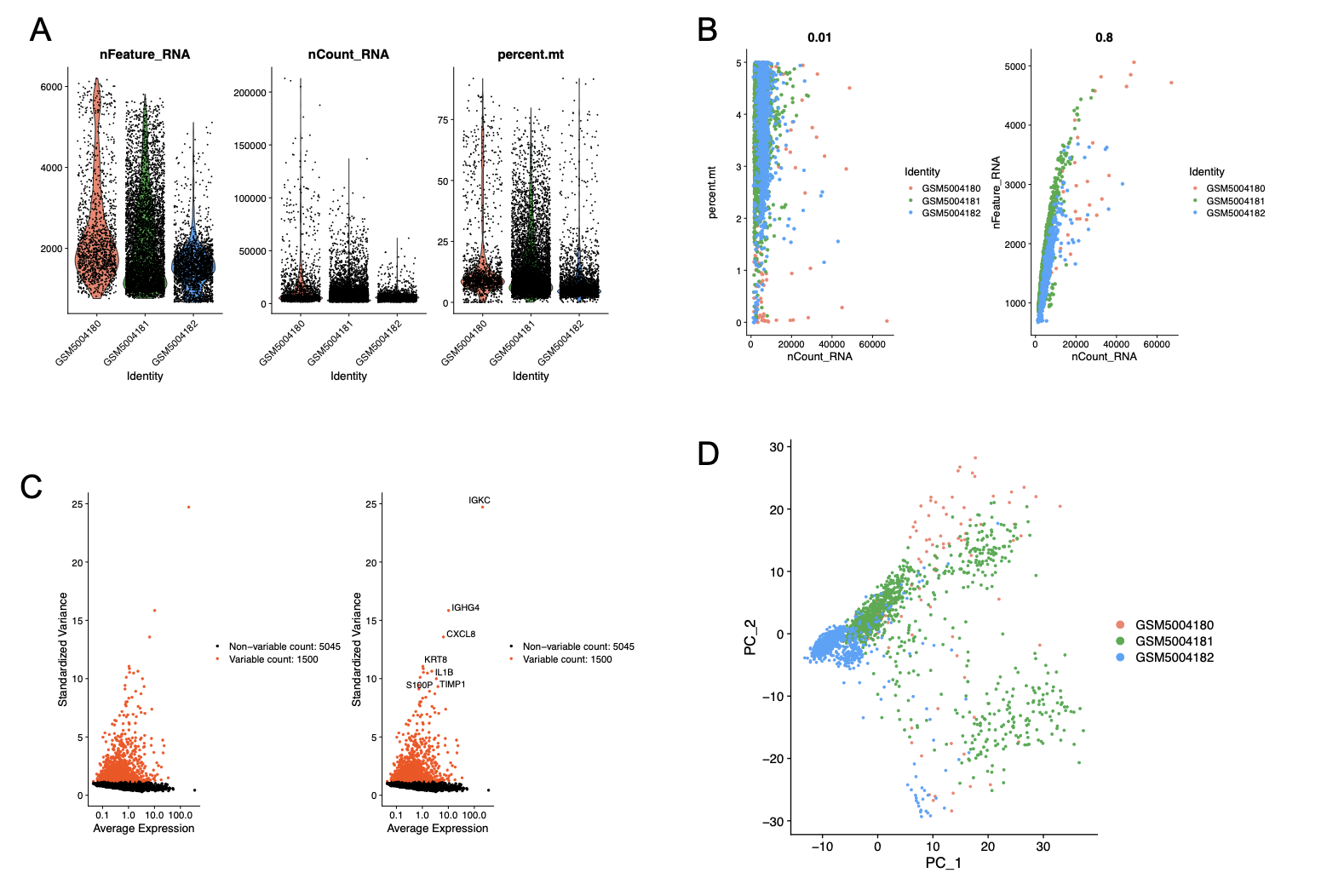

Supplement: Supplementary Figure 2 — Quality control and normalization of scRNA-seq data, dimensionality reduction and cell trajectory analysis. (A) After quality control and normalization, a total of 2057 cells were screened for further analysis. (B) Correlation analysis between sequencing depth and mitochondrial gene sequences as well as total intracellular sequences. (C) A total of 5,045 genes were analyzed, of which 3,545 genes had small intercellular variation and 1,500 genes had large variation. (D) PCA based on scRNA-seq data. [file Image_2.tiff]

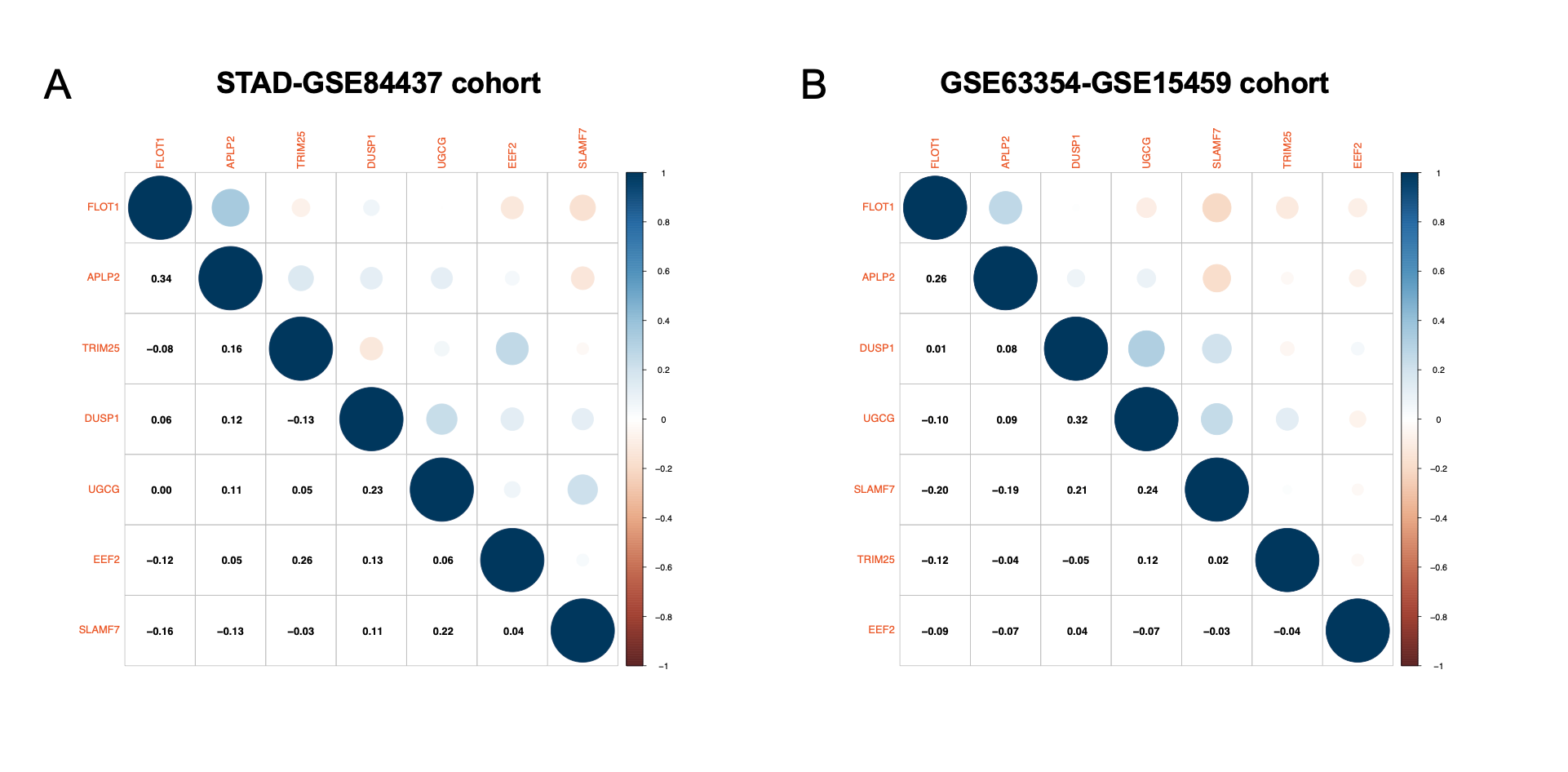

Supplement: Supplementary Figure 3 — (A) Spearman correlation analysis of model genes in STAD-GSE84437. (B) Spearman correlation analysis of model genes in the external validation cohort. [file Image_3.tiff]

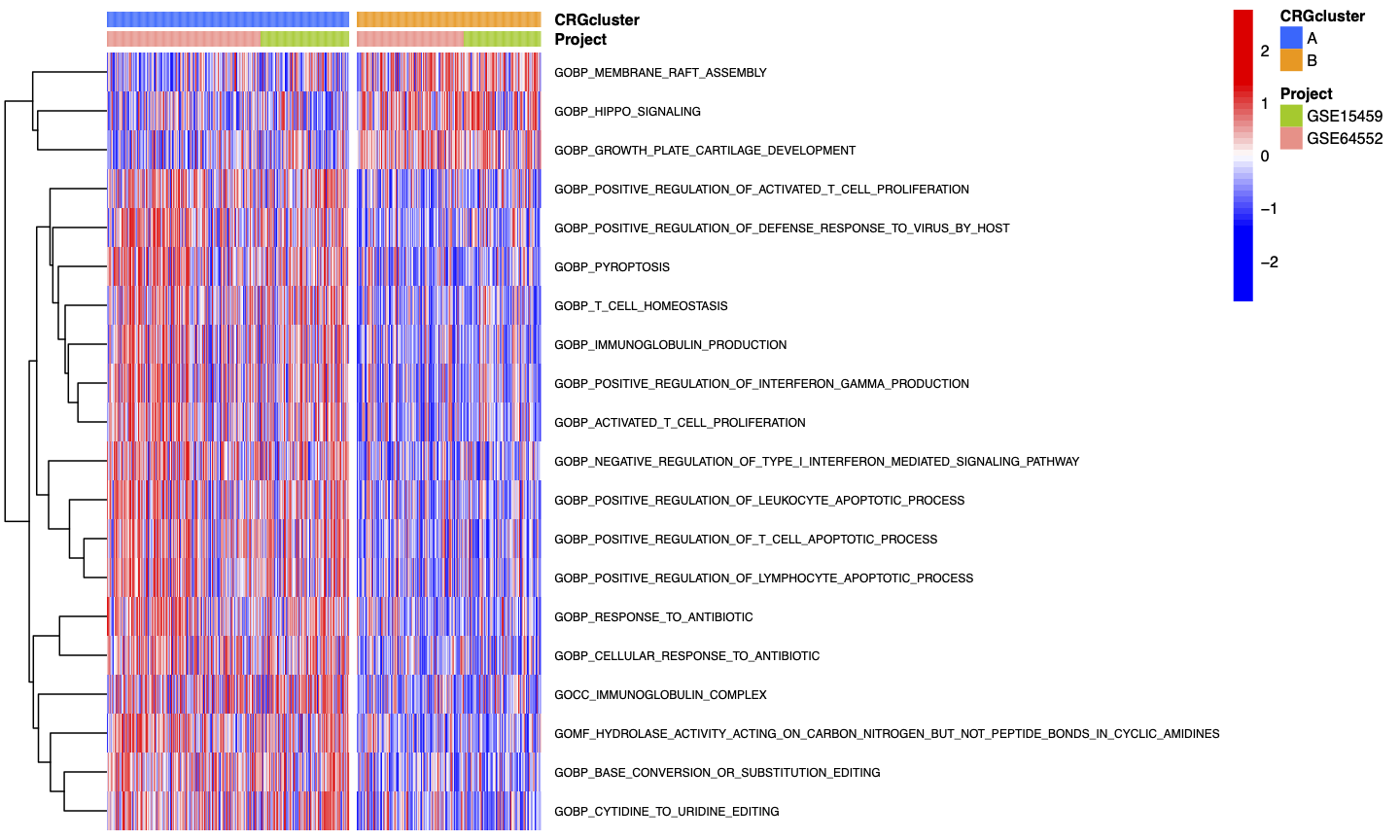

Supplement: Supplementary Figure 4 — Differential enrichment of GO annotations between low- and high-risk groups in the external validation cohort. [file Image_4.tiff]

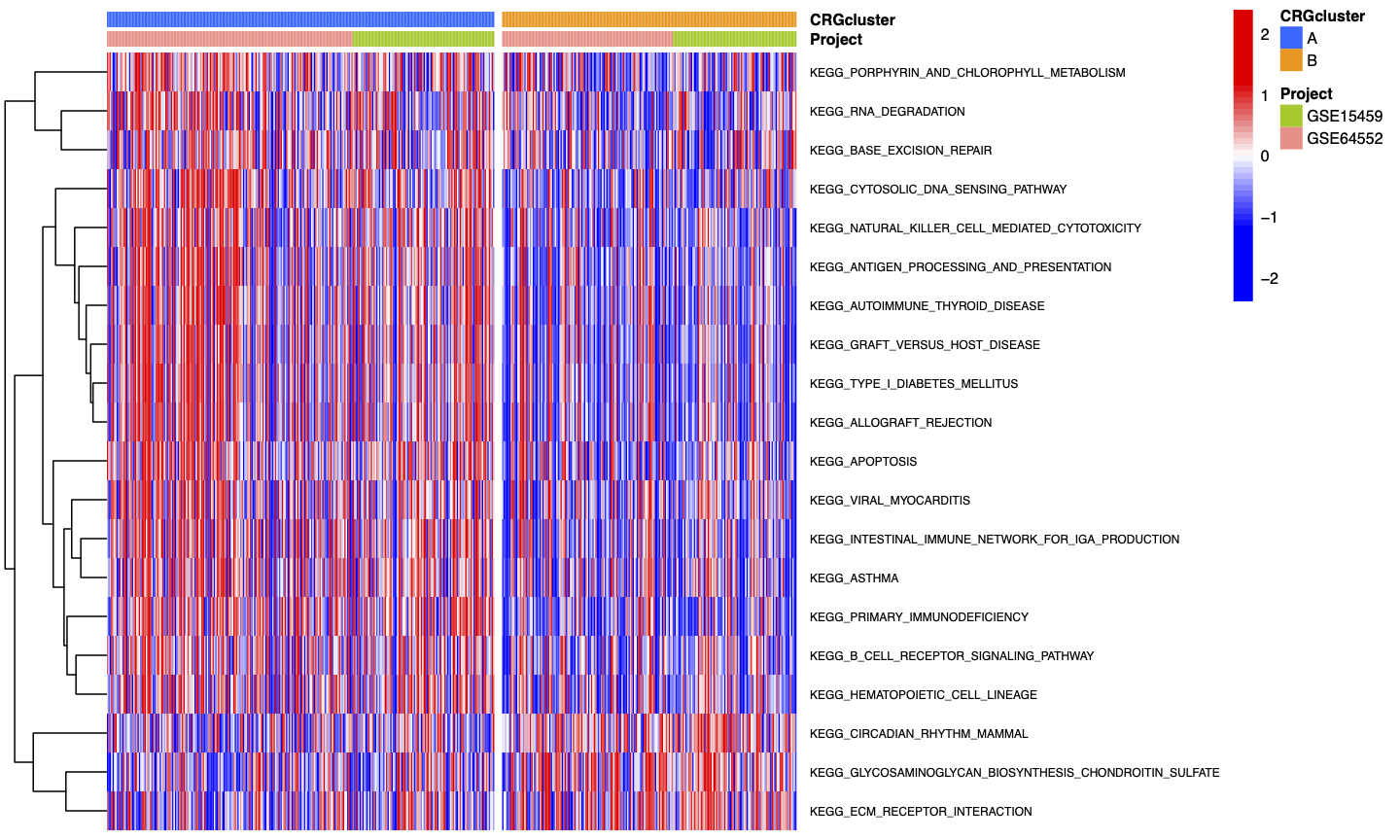

Supplement: Supplementary Figure 5 — Differential enrichment of KEGG pathways between low- and high-risk groups in the external validation cohort. [file Image_5.tiff]

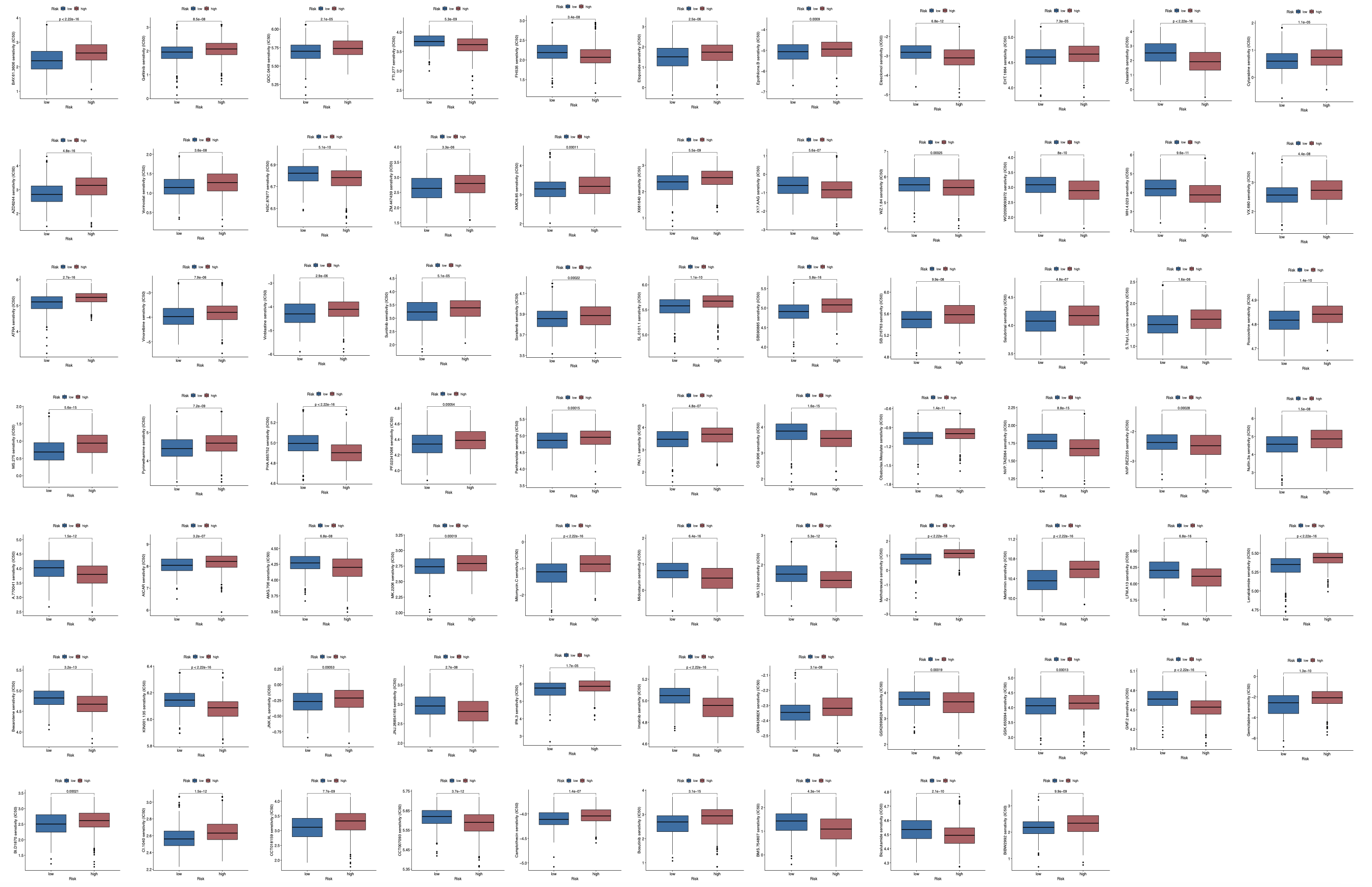

Supplement: Supplementary Figure 6 — Differences in drug sensitivity between low- and high-risk groups in STAD-GSE84437. [file Image_6.tiff]
