# Supplementary material for: Combining single-cell sequencing data to construct a prognostic signature to predict survival, immune microenvironment, and immunotherapy response in gastric cancer patients
Source: Front Immunol. 2022 Oct 10;13:1018413. doi: 10.3389/fimmu.2022.1018413 (PMC9589350; doi:10.3389/fimmu.2022.1018413)
Supplement: Supplementary file 7 [file Table_1.docx]

Supplementary Table .1 GEO Datasets Summary

| Accession number | Platform | Sample size |
| --- | --- | --- |
| GSE84437 | GPL6947 | 433 |
| GSE62254 | GPL570 | 300 |
| GSE15459 | GPL570 | 200 |
| GSE163558 | GOL24676 | 10 |
